# Narcissism, Bullying, and Social Dominance in Youth: A Longitudinal Analysis

**DOI:** 10.1007/s10802-015-9974-1

**Published:** 2015-02-03

**Authors:** Albert Reijntjes, Marjolijn Vermande, Sander Thomaes, Frits Goossens, Tjeert Olthof, Liesbeth Aleva, Matty Van der Meulen

**Affiliations:** 1 Department of Pedagogical and Educational Sciences, Utrecht University, PO Box 80150, Utrecht, The Netherlands; 2 University of Southampton, Southampton, UK; 3 Free University of Amsterdam, Amsterdam, The Netherlands; 4 Department of Developmental Psychological, Utrecht University, Utrecht, The Netherlands; 5 Groningen University, Groningen, The Netherlands

**Keywords:** Narcissism, Bullying, Social dominance, Joint trajectory analysis, Gender differences

## Abstract

A few previous studies have shown that narcissistic traits in youth are positively associated with bullying. However, research examining the developmental relationship between narcissism and bullying is lacking. Moreover, it is unclear whether narcissists constitute a homogeneous group and whether the bullying of narcissistic youth results in establishing social dominance over peers. The present work addresses these gaps. Children (*N* = 393; *M*_age_ = 10.3; 51 % girls) were followed during the last 3 years of primary school. Person-centered analyses were used to examine whether groups with distinct developmental trajectories for narcissism and two bullying forms (direct and indirect) can be identified, and how these trajectories are related. Multiple groups emerged for all constructs examined. For girls, higher narcissism was neither related to more intense bullying, nor to higher social dominance. In contrast, highly narcissistic boys were more likely than their peers to show elevated direct bullying, and in particular elevated indirect bullying. Hence, high narcissism is a risk factor for bullying in boys, but not in girls. However, narcissism is not always accompanied by high bullying, given that many boys on the high bullying trajectories were not high in narcissism. Results show that among narcissistic youth only those who engage in high levels of bullying are high in social dominance.

**Electronic supplementary material:**

The online version of this article (doi:10.1007/s10802-015-9974-1) contains supplementary material, which is available to authorized users.

During the past decades, researchers have increasingly acknowledged that bullying is a strategic attempt to acquire a central, powerful and dominant position in the peer group (e.g., Olthof et al. 2011; Salmivalli and Peets 2009). For instance, Farrington (1993) observed that when asked “why do you bully?”, the most frequently reported answers are “to feel powerful” and “to look cool”. Moreover, in early adolescence bullies score significantly higher on status, power, and prestige goals than do their peers (Sijtsema et al. 2009).

The motivation of bullies to gain power, dominance, and prestige over others suggests that elevated narcissism might be a contributing factor. Narcissism is a dispositional trait that involves a sense of entitlement of privileged status over others, the belief that one is unique and more important than others, and an excessive need for approval and admiration from others to feed the grandiose - but ultimately vulnerable - self (Miller et al. 2007; Morf and Rhodewalt 2001). Similar to adults, youth with narcissistic traits often display rather aversive interpersonal behavior, such as arrogance, lack of empathy, exploitativeness and aggression (Morf and Rhodewalt 2001; Thomaes and Brummelman 2015).

According to the self-regulatory model of Morf and Rhodewalt (2001), narcissistic individuals use several techniques to maintain or enhance their inflated self-esteem. For instance, when faced with criticism, they often react aggressively in an attempt to restore their self-esteem. With regard to bullying, Salmivalli (2001) posited that the exploitativeness and lack of empathy that figure prominently in narcissists may lead to aggression being employed instrumentally to foster their grandiose self-views.

During the past decade, a few studies have examined the link between narcissistic features and bullying in youth. Taken together, findings support Salmivalli’s (2001) hypothesis. For instance, in an inpatient sample of youth aged 10–15, Stellwagen and Kerig (2013) found that psychopathy-linked narcissism (i.e., the grandiose self-perceptions and sense of entitlement characteristic of youth with psychopathic traits) was concurrently positively linked with scores for (ringleader) bullying. Similarly, Ang and colleagues (Ang et al. 2010) showed that narcissistic exploitativeness in Asian youth was concurrently positively associated, albeit weakly, with bullying. Moreover, longitudinal work among Greek-Cypriot adolescents aged 12–14 has demonstrated that bullying was higher and more stable among those scoring higher on narcissism at baseline (Fanti and Kimonis 2012). In a recent prospective study, Fanti and Henrich (2015) found that narcissistic children with low general self-esteem are in particular likely to bully.

Notwithstanding the merits of these studies, important research gaps remain. First, except for the study of Fanti and colleagues, there is a paucity of longitudinal research examining the link between narcissism and bullying. Consequently, also because Fanti and coworkers only assessed narcissism once (at baseline), the stability of the core constructs over time is largely unknown. Moreover, the dynamic, longitudinal relationship between narcissism and bullying remains to be investigated.

Second, previous work has almost exclusively employed a variable-centered approach. A significant drawback of this approach is that participants are treated as one homogeneous group in terms of how the predictors operate on the outcomes (Laursen and Hoff 2006). Importantly, in the case of distinct subgroups of bullies or narcissists (e.g., when a summary statistic such as a correlation does not equally apply to all participants), mean-level parameters may not describe any subgroup validly (Von Eye and Bogat 2006), and they are often least applicable to children with the most extreme scores. Moreover, when the potential heterogeneity of narcissism and bullying in this respect is taken into account, interventions can be fine-tuned for specific groups of children. At his point, it should be noted that several studies have shown that different trajectories of bullying behavior exist (e.g., high and medium) that differ in their functioning and development (e.g., Pepler et al. 2008; Reijntjes et al. 2013b). Although Fanti and Henrich (2015) distinguished between bullies and “uninvolved” children, they did not distinguish between potentially different bullying trajectory subgroups, and narcissism was treated as a continuous variable.

Third, studies examining the link between narcissism and bullying have used instruments that do not tap all facets of the narcissism construct. Specifically, the Antisocial Process Screening Device (APSD), employed by Stellwagen and Kerig (2013) as well as Fanti and Kimonis (2012), assesses narcissistic behavior that tends to co-occur with psychopathic traits, but not narcissistic cognitions and feelings (e.g., feelings of entitlement) that are at the core of the narcissism construct. For this reason, researchers using the APSD typically refer to the measured construct as “psychopathy-linked” narcissism (for differences between this construct and narcissism, see Thomaes and Brummelman 2015). In a similar vein, Ang et al. (2010) only used the “Exploitativeness” subscale of the Narcissistic Personality Questionnaire for Children-Revised (NPQC-R). In the present study, the Childhood Narcissism Scale (CNS; Thomaes et al. 2008) was employed. This widely used, comprehensive and psychometrically sound measure indexes narcissism as a general construct, and is well validated in Dutch samples of youth.

Fourth, to the extent that narcissistic children high in bullying pursue social dominance and power, no study has examined whether they are successful in this regard. As in resource control theory (RCT; Hawley 1999), we construe social dominance as competitive superiority, which is an aspect of relationship asymmetry. Social dominance is indexed by resource control; i.e., having access to desirable, but scarce social and material resources (Hawley 1999).

The present three-wave study that followed children from late childhood into early adolescence addressed these limitations by examining the relations between narcissism and bullying as they unfold over time. In so doing, we distinguished between direct and indirect bullying. Direct bullying pertains to behaviors in which the victim is overtly harassed (e.g., physically, verbally), while indirect bullying refers to behaviors that do not directly confront the victim (e.g., gossiping). An important reason to make this distinction is that both forms may be differentially linked to both gender and narcissism. For instance, boys are more inclined to engage in direct forms of aggression than girls, whereas both genders are about equally likely to display indirect forms of aggression (Card et al. 2008). Moreover, it has been argued that for narcissistic youth the use of indirect, relational aggression may be more effective than direct aggression in terms of acquiring and preserving a dominant position in the peer group (Golmaryami and Barry 2010). We therefore wanted to examine whether narcissistic youth differ in the extent to which they enact both forms of bullying. We studied children in this developmental period because during preadolescence the formation of positive peer relations is an essential developmental task (Hartup 1996), and concerns about social status figure prominently (Fossati et al. 2010; Salmivalli 2001).

To capture potential subgroup differences in the strength and form of the association between the constructs examined, person-centered analyses were employed (Nagin 2005). Specifically, joint trajectory analyses were used to examine whether groups with distinct developmental trajectories of narcissism and bullying can be identified, and how these trajectories are related. This person-centered approach relates the longitudinal course of two constructs of interest (Nagin and Tremblay 2001), thereby comprehensively investigating their dynamic co-occurrence over time. We also examined the outcomes of bullying and narcissism in terms of social dominance. Specifically, after identifying joint trajectories of bullying and narcissism, we compared the joint trajectory groups on their resource control scores. For the reasons outlined above, as well as research showing that the link between narcissism and aggression may differ between boys and girls (e.g., Pauletti et al. 2012), we also examined potential gender differences.

We expected to find at least two different developmental trajectories for both narcissism and the two types of bullying, including a high and a low trajectory. We also expected that only a relatively small group of participants, predominantly boys, would engage in consistently high levels of bullying. Similarly, given that the narcissistic traits in youth in the general population are normally distributed, although somewhat positively skewed (Thomaes and Brummelman 2015), we also expected that relatively few children would be consistently high in narcissism. Moreover, we hypothesized that children following the highest narcissism trajectory would be more likely than their peers to simultaneously follow a higher bullying trajectory. Finally, we expected that social dominance would be highest for children displaying both high bullying and high narcissism.

## Method

### Participants

Participants were 393 children (51 % girls) from 12 elementary schools throughout the Netherlands. The children were followed during their last 3 years at elementary school. At the start of the study in 2006 (T_1_), all participants were in fourth grade (*M*_age_ = 10.3; *SD* = 0.5). There was no school transition during this period, and almost all children remained in the same classroom with the same peers. Participation rates within classroom were very similar across years. Although SES was not formally assessed, the sample included pupils from a wide range of social backgrounds. Parents received a letter in which they were informed about the purpose of the study. They could either provide passive consent for their child’s participation by not communicating further with the researchers (96 %), or refuse by returning a preprinted objection form (4 %). Parents and children could withdraw from the study at any time. All children provided their own assent. We also obtained IRB approval and permission from the schools. The large majority (83 %) of the children was Caucasian (native Dutch). Other groups represented were pupils with at least one parent originating from Turkey, Morocco, Surinam, or another European country.

At T_2_, data were available for 371 participants (94.4 % of the original sample). At T_3_, the sample contained 336 participants (85.5 %). Attrition was mainly due to participants moving to other, non-participating schools. Children not participating at T_2_ and/or T_3_ did not differ from children with complete data in their scores on relevant variables at T_1_ (*p*’s > 0.10).

### Procedure

Children’s self-reported narcissism scores were obtained during a classroom session run by trained research assistants. Teachers rated children’s resource control at their own convenience. The peer nominations were collected during an interview by a research assistant in a quiet room at the school grounds. Children could discontinue their participation at any time, but no child did. To minimize interviewer effects, research assistants were extensively trained, written research protocols were employed, and standardized interviews were laptop administered.

When providing peer nominations for bullying, children used a list containing the names of their classmates. The number of nominations was unlimited. Research on peer sociometric status has shown that, relative to the limited nominations approach, the unlimited nominations procedure yields a more reliable and valid assessment (Terry 2000). We consider it likely that this will also be the case for bullying.

Children could only nominate children from their own classroom, and not themselves. Mixed-sex nominations were used and nominations were conducted within classrooms rather than within grade. Although in early adolescence gender segregation is salient, in their study examining peer sociometric nominations (i.e., “like most” and “like least”) in sixth graders, Poulin and Dishion (2008) observed that including nominations from other-sex classmates improved the predictive validity of the sociometric measure. Moreover, for children confined to a stable classroom in which they mainly interact with their classmates and not much with other grademates (which is the case in the Netherlands), restricting the voting population to the classroom peers did not affect the predictive validity of the measure.

### Measures

#### Narcissism

The Childhood Narcissism Scale (CNS; Thomaes et al. 2008) is a 10-item self-report measure that indexes trait narcissism in youth aged 8 and older. The CNS has a one-factor structure and was developed to measure narcissism as a general construct, without distinguishing between more narrowly defined dimensions or facets such as adaptive versus maladaptive narcissism. Previous research (Thomaes et al. 2008) has shown that CNS scores have both adaptive (agentic interpersonal orientation) and maladaptive correlates (exploitativeness). Using a large sample (*N* = 1020), a single-factor model was tested in MPlus using confirmatory factor analysis (Thomaes et al. 2008). All factor loadings were freely estimated and no residual correlations between items were allowed. Several measures of model fit indicated that a single-factor model provided a good fit to the data. Specifically, RMSEA = 0.05; CFI = 0.95; NFI = 0.94. Standardized factor loadings ranged from 0.47 to 0.64 and all were significant. The internal consistency and the test-retest stability of the instrument are good (see Thomaes et al. 2008).

Sample items are “*I am a great example for other kids to follow*,” and “*I love showing all the things I can do*.” Items are rated on a scale ranging from 0 (*not at all true*) to 3 (*completely true*). In the present study, sum scores were used. Research shows that childhood narcissism has largely similar correlates and outcomes as adult narcissism. For instance, CNS scores are positively associated with self-appraised superiority, but only weakly with self-esteem (see Thomaes et al. 2008; Thomaes and Brummelman 2015). Moreover, attesting to construct validity, scores on the CNS are positively associated with self-esteem contingency, agentic but not communal social goals, psychopathic and Machiavellian personality traits, aggression, and negatively with empathy (Thomaes et al. 2008). Cronbach’s alpha was adequate at all assessment points (>0.75 for both genders).

#### Bullying

The bullying role nomination procedure (BRNP; Olthof et al. 2011) was used. The BRNP is a comprehensive measure that indexes both direct forms of bullying (e.g., hitting, damaging belongings of others, calling names) and indirect forms of bullying (e.g., gossiping, spreading rumors, stirring others up to exclude someone). Previous work (Olthof et al. 2011; Reijntjes et al. 2013a, b) has shown that bullying as indexed by the BRNP is associated in theoretically meaningful ways with peer-nominated perceived popularity, peer-rated likeability, and self-perceived social competence.

To avoid potential interpretation differences of the term bullying, children first received an elaborate description of the concept, in which its three core features were explained: intent to harm, repetition over time, and a patent power difference between perpetrator and victim (Salmivalli and Peets 2009). Children were told that bullying can occur in a number of ways and differs from a quarrel or fight between two equally strong peers.

Subsequently, five specific types of bullying were described (i.e., physical, possession-related, verbal, direct relational, and indirect relational) and nominations were obtained by asking “*Do you know which classmates carry out that particular form of bullying?*”, and “*If so, could you give us their names?*” Continuous scores for both direct and indirect bullying were computed within classrooms by dividing the total number of nominations by the number of nominators minus 1 (the participant himself; see Goossens et al. 2006; Kärnä et al. 2011). Scores were then standardized within classes to take differences between nominating groups into account. A total bullying score was also calculated by summing the scores for the two forms of bullying. Analyses were performed for the two bullying forms separately and for the total bullying score.

#### Resource Control

This construct indexes social dominance and involves having access to scarce, desirable material and social resources (Hawley 1999). Teachers rated participants on six items, on a scale ranging from 0 (*never or almost never*) to 4 (*very often*). Sample items are: “*To what extent is this child usually at the center of attention in a group of children*”?; and “*To what extent does this child usually get what it wants*?” The ratings were averaged. The scale showed high internal consistency (Cronbach’s alpha > 0.90 at all three assessment points). To take differences between teachers into account, the scores were standardized within each class.

### Plan of Analyses

We first present descriptive data and correlational analyses. Next, the person-centered analyses proceeded in three steps. First, the group-based trajectory approach (Nagin 2005) was used to estimate separate models for the developmental trajectories of narcissism, the two forms of bullying, and total bullying. Using MPlus version 6.11 (Muthén and Muthén 2010), latent class growth analyses models (LCGA’s; Muthén and Muthén 2000) were employed. Missing data for participants who did not complete an entire measure (as opposed to individual items) and for those who did not complete one or two complete assessments were handled through full information maximum likelihood (FIML).

Latent class growth analysis uses an outcome variable measured at multiple time points to define a latent class model in which the latent classes correspond to different growth curves for that variable, thereby yielding clusters of individuals who follow distinct developmental trajectories. In the case of three assessment points, these trajectories are identified on the basis of two parameters; i.e., intercepts (starting values) and linear slopes. The proportion of individuals following each of the trajectories is estimated.

For narcissism, the two bullying forms, and total bullying a series of models was fitted, starting with a one-trajectory model and moving to a five-trajectory model. To make a well-founded decision regarding the optimal number of groups, several statistical indicators were used (as recommended by Nagin 2005), including the Bayesian Information Criterion (BIC), the Lo-Mendell-Rubin likelihood ratio test (LMR-LRT), and entropy.

Following Nagin (2005), we also examined whether for all groups the average posterior probability (*AvePPj*) exceeded 0.70. This signifies that, on average, the chance that children assigned to a particular trajectory group actually belong to that group is at least 0.70. Second, we examined whether the odds of correct classification (*OCC*_*j*_) were at least 5 for all groups. Finally, we compared the model estimated proportion of the population following a particular trajectory group (*π*_*j*_) with the corresponding proportion of the sample assigned to that trajectory (*P*_*j*_), with less discrepancy indicating better model fit.

After determining the best fitting trajectory models for the targeted variables separately, in the second step the joint trajectories for (a) narcissism and (b) each of the two bullying forms, as well as the total bullying score were estimated. Key outputs of a joint model are joint probabilities and two sets of conditional probabilities. Joint probabilities pertain to the proportion of children estimated to belong simultaneously to certain trajectory groups of both variable A and variable B (e.g., children who follow both the high narcissism trajectory as well as the high physical bullying trajectory). When *j* and *k* index the trajectory groups associated with bullying and narcissism, the joint probabilities are denoted by *π* ^*jk*^ and are provided as part of the output. Conditional probabilities pertain to the estimated probability of belonging to a specific trajectory group for variable A (e.g., high direct bullying) given membership of a specific trajectory group for variable B (e.g., high narcissism), and vice versa. These probabilities are denoted by *π* ^*j*|*k*^ *and π* ^*k*|*j*^ and are calculated as follows: ${\pi}^{j\left|k\right.}=\frac{\pi^{jk}}{\pi^k},$ with ${\pi}^k={\displaystyle {\sum}_j{\pi}^{jk},}\kern.3em k=1,\dots, K\kern.3em  and\kern.3em {\pi}^{k\left|j\right.}=\frac{\pi^{jk}}{\pi^j},$ with ${\pi}^j={\displaystyle {\sum}_j{\pi}^{jk},\kern1em j=1,\dots, J.}$

Importantly, conditional probabilities do not imply a time order relationship but reflect the probability of simultaneously following two trajectories during the same period. To evaluate differences between observed probabilities, we used a Bayesian model selection approach with (in)equality constraints between the parameters of interest (Klugkist et al. 2005). The results of the Bayesian approach are expressed in terms of posterior model probabilities (*PMP’*s), representing the probability that the specific model at hand receives most support from the data among a set of models (e.g., Model 1: probability A is larger than probability B, versus Model 2: probability A is equal to probability B). A model was considered to outperform another model when its *PMP* was at least 0.95 (Klugkist et al. 2005).

Finally, we examined how social dominance scores differed as a function of the joint effects of bullying and narcissism. Specifically, after identifying the joint trajectories of narcissism and total bullying score, we compared these joint groups on their mean resource control scores across the three waves. We also aimed to investigate how the joint trajectories of narcissism and the two different bullying forms separately are related to social dominance scores. However, examining how narcissism and each of the two bullying forms separately contribute to social dominance proved problematic. Specifically, when examining social dominance as a function of narcissism and direct (indirect) bullying, one should control for the effect of indirect (direct) bullying. Given our approach, which yields distinct trajectory groups (latent classes) for both types of bullying, this requires controlling for class membership of indirect bullying when examining the effects of direct bullying (and vice versa). However, whereas controlling for a continuous covariate is possible, current software does not allow for controlling for (the probability of) latent class membership.

At this point, it should be noted that an additional joint trajectory analysis showed that almost all participants who were assigned to the high (medium, low) direct bullying trajectory group, simultaneously belonged to the high (medium, low) indirect bullying trajectory group. This finding indicates that an observation of high (low) indirect bullying is almost synonymous with an observation of high (low) direct bullying, and vice versa. Given that both bullying forms were strongly interwoven, we decided to examine how the joint trajectories of narcissism and both bullying behaviors combined (i.e., total bullying) relate to social dominance (resource control) scores.

## Results

### Preliminary Analyses

Table 1 displays the means and standard deviations for all variables at the three assessment points. Across waves, boys scored higher on narcissism than did girls (*p*’s < 0.05), although the differences were small (Cohen *d*’s < 0.25). No gender differences were observed for resource control (*p*’s > 0.20). For both forms of bullying, boys consistently scored higher than girls, except for indirect bullying at T_3_. Gender differences were largest for direct bullying. Across gender, the two forms of bullying were substantially correlated at all three assessment points (*r*’s > 0.65; *p*’s < 0.001; see correlation Tables in the Electronic supplementary material).

**Table 1:** Means and standard deviations of narcissism, bullying, and resource control

	Boys		Girls		Total	
	Mean*D*	*SD*	Mean	*SD*	Mean	*SD*
Narcissism						
Wave1	9.11_1_	4.75	8.16_2_	4.83	8.62	4.81
Wave2	8.40_1_	4.61	7.46_2_	4.61	7.91	4.63
Wave3	8.91_1_	4.35	7.88_2_	4.47	8.37	4.44
Direct bullying						
Wave1	0.47 _1,a_	0.53	0.12 _2_	0.21	0.29 _a_	0.44
Wave2	0.39 _1,b_	0.50	0.11 _2_	0.21	0.25 _b_	0.41
Wave3	0.40 _1,b_	0.54	0.11 _2_	0.16	0.25 _b_	0.41
Indirect bullying						
Wave1	.10_1_	0.12	.05_2_	0.07	0.07	0.10
Wave2	.09_1_	0.12	.05_2_	0.10	0.07	0.11
Wave3	0.07	0.09	0.08	0.11	0.07	0.10
Total Bullying						
Wave1	.57_1,a_	0.64	.17_2_	0.27	0.37 _a_	0.52
Wave2	0.48 _1,b_	0.60	.16_2_	0.28	0.31 _b_	0.49
Wave3	.47_1,b_	0.61	.19_2_	0.25	0.33 _b_	0.48
Resource control						
Wave 1	1.82	0.99	1.55	1.00	1.68	1.01
Wave 2	2.05	1.25	1.96	1.07	2.00	1.16
Wave 3	2.05	0.80	1.99	0.75	2.01	0.77

Note. Different subscripts (*numbers*) in the same row denote significant gender differences. Different subscripts in the same column (*letters*) denote significant differences over time (*p*’s < 0.05)

Repeated measures ANOVA analyses revealed substantial stability for all constructs over time. For narcissism and resource control, the mean score did not change over time. Scores for bullying were also quite stable over time. Only a decrease in direct and total bullying was observed from T_1_ to T_2_, and this change only applied to boys.

At all three time points, the linkage between narcissism and resource control was low (*r*’s < 0.13), although significant at T_1_ and T_3_ (see Electronic supplementary material). Similarly, at all three time points scores for narcissism and each of the two bullying forms and total bullying were only weakly related (*r*’s < 0.18), albeit significantly in several instances. In contrast, across time substantial linkages were found between resource control and both bullying types, as well as total bullying (*r*’s ranging from 0.39 to 0.49; *p*’s < 0.01).

### LCGA Analyses

Separate trajectory analyses were performed for narcissism, direct bullying, indirect bullying, and the total bullying score. Participants were assigned to the trajectory group for which they showed the highest posterior probability.

#### Narcissism

The statistical indicators provided most support for a three-group model. Specifically, when moving from a two-group to a three-group model, entropy increased from 0.68 to 0.70, the LMR-statistic was significant, and the BIC value decreased from 6230.8 to 6189.3. However, when moving to a four-group model, entropy decreased to 0.61, the LMR- statistic was not significant, and the BIC value increased to 6200.3. Importantly, the fit indices for the three-group model were good (*AvePP j*’s > 0.83; *OCC j*’s > 5; differences between *Pj* and *π*_*j*_ less than 2 %, entropy = 0.70).

As depicted in Figure 1 (see Electronic supplementary material), the largest group (*n* = 184; 46.8 % of the sample) displayed stable medium narcissism scores (intercept (I) = 9.60, *p* < 0.001; slope (S) = 0.27, *p* > 0.20). Children in the second largest group (*n* = 171; 43.5 %) showed consistently low scores (*I* = 5.46, *p* < 0.001; *S* = −0.36, *p* > 0.20). Children in the third and smallest group (*n* = 37; 9.4 %) displayed the highest scores that were stable over time (*I* = 15.56, *p* < 0.001; *S* = −0.21; *p* > 0.20). Boys were overrepresented in the high and medium trajectory groups (56.8 % and 55.4 %, respectively), and underrepresented in the lowest group (40.9 %): *χ* ^2^(2) = 8.38, *p* < 0.02.

#### Direct Bullying

A four-group model was selected as fitting the data best. When moving from a three-group to a four-group model, the LMR-statistic was significant, and the BIC value decreased from 2318.7 to 2290.3. However, when moving to a five-group model, entropy decreased from 0.78 to 0.74, the LMR-statistic was not significant, and the BIC value increased to 2307.7. The fit indices for the four-group model were good (*AvePP j*’s > 0.80; *OCC j*’s > 5; differences between *Pj* and *π*_*j*_ less than 2 %; entropy = 0.78).

As depicted in Figure 1, children in the largest group (*n* = 148; 37.6 % of the sample) displayed stable low scores (*I* = −0.71, *p* < 0.001; *S* = 0.03, *p* > 0.30). Children in the second largest group (*n* = 115; 29.4 %) engaged in stable, average levels of direct bullying (I = −0.09, *p* > 0.20; S = 0.08, *p* > 0.20). The scores of children in the third largest group (*n* = 92; 23.5 %) were medium, and they did not change over time (*I* = 0.74, *p* < 0.01; *S* = −0.001, *p* > 0.20). The smallest group (*n* = 37; 9.5 %) consisted of those children displaying the highest scores, which were stable over time (*I* = 1.55, *p* < 0.01; *S* = 0.08, *p* > 0.10). Boys were overrepresented in both the high and medium trajectory groups (89.2 and 69.6 %), and underrepresented in the average and low direct bullying trajectory groups (41.7 and 24.1 %): *χ* ^2^(3) = 57.60, *p* < 0.001.

#### Indirect Bullying

A three-group model fitted the data best. When moving from a two-group to a three-group model, the LMR-statistic was significant, and the BIC value decreased from 2364.0 to 2347.3. However, when moving to a four-group model, entropy decreased from 0.76 to 0.73, the LMR-statistic was not significant, and the BIC value increased to 2391.3. The fit indices for the three-group model were adequate (*AvePP j*’s > 0.75; *OCC j*’s > 5; differences between *Pj* and *π*_*j*_ less than 3 %; entropy = 0.76). Participants in the largest group (*n* = 260; 66.3 %) displayed the lowest scores, which slightly increased over time (*I* = −0.43, *p* < 0.001; *S* = 0.08, *p* < 0.05; see Figure 1). Children in the second largest group (*n* = 67; 17.1 %) engaged in the highest levels of indirect bullying, which did not change over time (*I* = 1.24, *p* < 0.001; *S* = 0.02, *p* > 0.20). The third group was almost as large (*n* = 64; 16.6), and children in this group showed stable medium scores (*I* = 0.61, *p* < 0.001; *S* = −0.23, *p* > 0.10). Boys were overrepresented in the high and medium trajectory groups (62.7 and 62.5 %), but underrepresented in the low trajectory group (42.7 %): *χ* ^2^(2) = 13.81, *p* < 0.001.

#### Total Bullying

A four-group model was selected. When moving from a three-group to a four-group model, the LMR-statistic was significant, and the BIC value decreased from 2320.4 to 2307.5. However, when going to a five-group model, the BIC value increased to 2315.0, and the LMR-statistic was not significant. The fit indices for the four-group model were good (*AvePP j*’s > 0.75; *OCC j*’s > 5; differences between *Pj* and *π*_*j*_ less than 2 %; entropy = 0.74). Children in the largest group (*n* = 146; 37.2 %) displayed stable low scores (*I* = −0.77, *p* < 0.001; *S* = 0.01, *p* > 0.20; see Figure 1). Those assigned to the second largest group (*n* = 109; 27.8 %) scored average on total bullying, and their scores did not change over time (*I* = −0.08; *p* > 0.20; *S* = 0.03; *p* > 0.10). Children in the third largest group (*n* = 94; 24.0 %) showed stable scores that were medium in magnitude (*I* = 0.72, *p* < 0.001; S = −0.03, *p* > 0.15). The smallest group (*n* = 43; 11.0 %) consisted of participants displaying stable high scores (*I* = 1.55, *p* < 0.01; *S* = 0.07, *p* > 0.10). Boys were overrepresented in the high and medium trajectory groups (86.0 and 67.0 %), and underrepresented in the average and low trajectory groups (40.4 and 34.5 %): *χ* ^2^(3) = 49.88, *p* < 0.001.

### Primary Analyses

The joint analyses were initially performed for boys and girls combined. That is, in all instances we first determined for the entire sample the joint probabilities *π* ^*jk*^ of belonging to two different trajectory groups simultaneously (e.g., high narcissism and high indirect bullying). Results showed that these joint probabilities differed substantially across both genders, and that girls were underrepresented in both the high narcissism trajectory group and the two high bullying trajectory groups. Given these observations, combining both genders when presenting the joint and conditional probabilities would have concealed important differences between boys and girls in terms of the dynamic overlap between narcissism and bullying. We therefore considered it more appropriate to perform these analyses separately by gender, such that for boys and girls distinct joint and conditional probabilities were computed and compared using the Bayesian approach outlined above (see Plan of Analyses).

#### Joint Trajectories of Narcissism and Direct Bullying

The top panel of Table 2 shows the joint probabilities of trajectory membership for narcissism and direct bullying, for boys and girls separately. In this part of the Table, probabilities across all cells sum to 1. The largest subgroup of boys scored medium on both narcissism and direct bullying (*n* = 46; 24 %), while most girls scored low on both constructs (*n* = 62; 31 %). A relatively small subgroup of the boys (*n* = 8; 4 %), but no girl, scored high on both constructs simultaneously.

**Table 2:** Joint and conditional probabilities of narcissism and direct bullying trajectories

Narcissism	Direct bullying			
	High	Medium	Average	Low
Joint probability of trajectory group membership ^a^				
High	0.04(*n* = 8)/ –	0.05(*n* = 9)/ –	– / **0.04(** ***n*** **= 8)**	0.02(*n* = 4)/**0.05(** ***n*** **= 9)**
Medium	0.06(*n* = 11)/**0.02(** ***n*** **= 3)**	0.24(*n* = 46)/**0.02(** ***n*** **= 13)**	0.12(*n* = 24)/**0.10(n = 20)**	0.12(*n* = 24)/**0.19(** ***n*** **= 37)**
Low	0.06(*n* = 12)/**0.01(** ***n*** **= 2)**	0.08(*n* = 16)/**0.05(** ***n*** **= 9)**	0.11(*n* = 22)/**0.18(n = 35)**	0.09(*n* = 17)/**0.31(** ***n*** **= 62)**
	Direct bullying			
	High	Medium	Average	Low
Probabilities of direct bullying conditioned on narcissism ^b^				
High narcissism (*N*_*b*_ = 21; N_g_ = 17)	0.38(*n* = 8)/ –	0.43(*n* = 9)/ –	– / **0.47(** ***n*** **= 8)**	0.19(*n* = 4)/**0.53(** ***n*** **= 9)**
Medium narcissism (*N*_*b*_ = 105; *N*_*g*_ = 73)	0.10(*n* = 11)/**0.04(** ***n*** **= 3)**	0.44(*n* = 46)/**0.18(** ***n*** **= 13)**	0.23(*n* = 24)/**0.27(** ***n*** **= 20)**	0.23(*n* = 24)/**0.51(** ***n*** **= 37)**
Low narcissism (*N*_*b*_ = 67; *N*_*g*_ = 108)	0.18(*n* = 12)/**0.02(** ***n*** **= 2)**	0.24(*n* = 16)/**0.08(** ***n*** **= 9)**	0.33(*n* = 22)/**0.32(** ***n*** **= 35)**	0.25(*n* = 17)/**0.57(** ***n*** **= 62)**
	Narcissism			
	High	Medium	Low	
Probabilities of narcissism conditioned on direct bullying ^b^				
High bullying (*N*_*b*_ = 31; *N*_*g*_ = 5)	0.26 (*n* = 8) / –	0.35 (*n* = 11) / **0.60 (** ***n*** **= 3)**	0.39 (*n* = 12) / **0.40(** ***n*** **= 2)**	
Medium bull. (*N*_*b*_ = 71; *N*_*g*_ = 22)	0.13 (*n* = 9) / –	0.65 (*n* = 46) / 0**.59 (** ***n*** **= 13)**	0.22 (*n* = 16) / **0.41(** ***n*** **= 9)**	
Average bull. (*N*_*b*_ = 46; *N*_*g*_ = 63)	– / **0.13 (** ***n*** **= 8)**	0.52 (*n* = 24) / **0.32 (** ***n*** **= 20)**	0.48 (*n* = 22) / **0.55 (** ***n*** **= 35)**	
Low bullying (*N*_*b*_ = 45; *N*_*g*_ = 108)	0.09 (*n* = 4) / 0**.09 (** ***n*** **= 9)**	0.53 (*n* = 24) **/ 0.34 (** ***n*** **= 37)**	0.38 (*n* = 17) / **0.53 (** ***n*** **= 62)**	

^a^ Cells sum to 1. ^b^ Rows sum to 1. Figures in bold pertain to girls

#### Conditional Probabilities of Direct Bullying Given Narcissism

The middle panel of Table 2 presents the likelihood of following one of the four direct bullying trajectories conditional on membership of a specific narcissism trajectory group. Boys on the high narcissism trajectory were much more likely to follow the high than the low bullying trajectory (probabilities 0.38 and 0.19, respectively; PMP > 0.95), whereas for boys following the low narcissism trajectory these probabilities were equally high (0.18 and 0.25, respectively). Moreover, highly narcissistic boys were substantially more likely than their peers medium or low in narcissism to follow the highest bullying trajectory (PMP’s > 0.95).

For girls, findings were markedly different. Specifically, girls in all three narcissism trajectory groups were far more likely to follow the low than the high bullying trajectory (PMP’s > 0.95). In fact, across the three narcissism trajectory groups, for girls the probability to follow the low bullying trajectory was highest, and the probability to follow the high bullying trajectory was lowest. Taken together, contrary to boys, results for girls indicate no systematic developmental overlap between narcissism and direct bullying.

#### Conditional Probabilities of Narcissism Given Direct Bullying

The bottom panel of Table 2 presents the likelihood of following one of the three narcissism trajectories conditional on membership of a specific direct bullying trajectory group. Boys on the highest bullying trajectory were about equally likely to follow the three narcissism trajectories (probabilities ranging from 0.26 to 0.39). This finding indicates high levels of direct bullying are also quite common among boys who are not high in narcissism. Whereas a substantial minority of the high bullying boys was also high in narcissism (probability 0.26), boys assigned to the other three bullying trajectories were much less likely to be high on narcissism (probabilities ranging from 0 to 0.13; PMP’s > 0.95). Hence, boys who do not score high on direct bullying are quite unlikely to be high on narcissism.

For girls, the two sets of conditional probabilities were more symmetrical. Specifically, for all four bullying groups the probability to belong to the high narcissism group was lowest (and even zero for the two highest bullying groups). Girls high or medium on bullying were most often assigned to the medium narcissism group, while girls in the average and low bullying group were most likely to be simultaneously low in narcissism.

#### Joint Trajectories of Narcissism and Indirect Bullying

Most boys were low in indirect bullying, and simultaneously medium or low in narcissism (28 and 26 %; see Table 3). The largest subgroup of girls scored low on both constructs (42 %). Twelve boys, but no girl, scored high on both narcissism and indirect bullying simultaneously.

**Table 3:** Joint and conditional probabilities of narcissism and indirect bullying trajectories

Narcissism	Indirect bullying		
	High	Medium	Low
Joint probability of trajectory group membership ^a^			
High	0.06(*n* = 12)/ –	0.01(*n* = 2) / **0.005 (** ***n*** **= 1)**	0.03(*n* = 6) / **0.07 (** ***n*** **= 14)**
Medium	0.08(*n* = 15)/ **0.03(n = 5)**	0.19(*n* = 37)/ **0.05 (** ***n*** **= 10)**	0.28(*n* = 55)/ **0.29 (** ***n*** **= 58)**
Low	0.06(*n* = 12)/ **0.02(n = 3)**	0.02(n = 4) / **0.12 (** ***n*** **= 23)**	0.26(*n* = 50)/ **0.42 (** ***n*** **= 84)**
	Indirect bullying		
	High	Medium	Low
Probabilities of indirect bullying conditioned on narcissism ^b^			
High narcissism (*N*_*b*_ = 20; *N*_*g*_ = 15)	0.60 (*n* = 12)/ –	0.10(*n* = 2) / **0.07 (n = 1)**	0.30(*n* = 6) / **0.93 (** ***n*** **= 14)**
Medium narcissism (*N*_*b*_ = 107; *N*_*g*_ =73)	0.14(*n* = 15) / **0.07(n = 5)**	0.35(*n* = 37)/ **0.14 (** ***n*** **= 10)**	0.51(*n* = 55)/ **0.79 (** ***n*** **= 58)**
Low narcissism (*N*_*b*_ = 64; *N*_*g*_ = 108)	0.19(*n* = 12)/ **0.03(** ***n*** **= 3)**	0.06(*n* = 4) / **0.21 (** ***n*** **= 23)**	0.75(*n* = 50)/ **0.76 (** ***n*** **= 84)**
	Narcissism		
	High	Medium	Low
Probabilities of narcissism conditioned on indirect bullying ^b^			
High indirect bullying (*N*_*b*_ = 39; *N*_*g*_ = 8)	0.31 (*n* = 12)/ –	0.38 (*n* = 15)/ **0.63 (** ***n*** **= 5)**	0.31 (*n* = 12)/ **0.37 (** ***n*** **= 3)**
Medium indirect bullying (*N*_*b*_ = 43; *N*_*g*_ = 34)	0.05(*n* = 2)/ **0.03(** ***n*** **= 1)**	0.86 (*n* = 37)/ **0.29 (** ***n*** **= 10)**	0.09(*n* = 4) **/ 0.68 (** ***n*** **= 23)**
Low indirect bullying (*N*_*b*_ = 111; *N*_*g*_ = 156)	0.05 (*n* = 6)/ **0.09(** ***n*** **= 14)**	0.50 (*n* = 55)/ **0.37(** ***n*** **= 58)**	0.45 (*n* = 50)/ **0.54 (** ***n*** **= 84)**

^a^ Cells sum to 1. ^b^ Rows sum to 1. Figures in bold pertain to girls

#### Conditional Probabilities of Indirect Bullying Given Narcissism

Boys on the high narcissism trajectory were much more likely (probability 0.60) than their peers medium or low in narcissism to follow the high bullying trajectory (probabilities 0.14 and 0.19; PMP’s > 0.95; see Table 3). Boys medium or low in narcissism were substantially more likely to follow the lowest than the highest indirect bullying trajectory (PMP’s > 0.95). Noteworthy, the probability of boys high on narcissism to follow the high indirect bullying trajectory (0.60) was substantially higher than was the probability of highly narcissistic boys to follow the high direct bullying trajectory (probability 0.38; PMP > 0.95).

Results for girls were markedly different. Across narcissism trajectory groups, girls rarely followed the high bullying trajectory (all probabilities < 0.10). Instead, in all three narcissism groups girls were most likely to follow the low indirect bullying trajectory (all probabilities > 0.75). Hence, among girls narcissism and indirect bullying were not related.

#### Conditional Probabilities of Narcissism Given Indirect Bullying

The bottom panel of Table 3 shows that boys on the highest indirect bullying trajectory were about equally likely to follow each of the three narcissism trajectories, indicating that high narcissism is one of multiple factors contributing to high indirect bullying. Compared to the high bullying boys, it was far less common for boys medium or low on bullying to be high in narcissism (probability 0.31, versus probability 0.05 for both groups; PMP’s > 0.95).

Girls in all indirect bullying trajectory groups were unlikely to simultaneously belong to the high narcissism group (all probabilities < 0.10). The eight girls high on indirect bullying were most likely to belong to the medium narcissism group, whereas the girls in the two other bullying groups were most often low in narcissism.

#### Single and Combined Effects of Narcissism and Total Bullying Score on Social Dominance Outcome Scores

The top panel of Table 4 displays the number of children assigned to each of the joint trajectories of narcissism and total bullying, for both genders separately. The largest subgroup of boys scored medium on both narcissism and total bullying (*n* = 37; 19 %), whereas the largest subgroup of girls scored low on both variables simultaneously (*n* = 51; 26 %). A small subgroup of the boys (*n* = 7; 3 %) scored high on both constructs, whereas no girl did so.

**Table 4:** Joint probabilities of narcissism and total bullying trajectories (upper panel) and mean resource control scores for the joint trajectory groups (bottom panel)

Narcissism	Total bullying			
	High	Medium	Average	Low
Joint probability of trajectory group membership ^a^				
High	0.03(*n* = 7)/ –	0.04(*n* = 8)/ **0.005(** ***n*** **= 1)**	0.01(*n* = 2)/ **0.03(** ***n*** **= 5)**	0.02(*n* = 4)/**0.05(** ***n*** **= 9)**
Medium	0.09(*n* = 18)/**0.02(** ***n*** **= 4)**	0.19(*n* = 37)/**0.08(** ***n*** **= 15)**	0.13(*n* = 25)/**0.14(** ***n*** **= 28)**	0.11(*n* = 21)/**0.18(** ***n*** **= 35)**
Low	0.06(*n* = 12)/**0.01(** ***n*** **= 2)**	0.09(*n* = 17)/**0.08(** ***n*** **= 16)**	0.09(*n* = 17)/**0.16(** ***n*** **= 32)**	0.13(*n* = 25)/**0.26(** ***n*** **= 51)**
	Total bullying			
	High	Medium	Average	Low
Mean score on resource control over time ^b^				
High narcissism (*N*_*b*_ = 21; *N*_*g*_ = 15)	0.93 (0.50)/ –	0.38 (0.77)/ **0.24 (N/A)**	−0.60 (0.64) /−**0.24(1.01)**	−0.57(1.05)/−**0.47 (0.81)**
Medium narcissism (*N*_*b*_ = 101; *N*_*g*_ = 74)	0.86 (0.68)/ **1.40 (0.32)**	0.40 (0.66)/ **0.75 (0.55)**	−0.25 (0.72) / **0.24 (0.67)**	−0.66 (0.73) /−**0.37 (0.62)**
Low narcissism (*N*_*b*_ = 71; *N*_*g*_ = 101)	0.56 (0.62)/**1.12(1.02)**	−0.12 (0.61)/ **0.47 (0.75)**	−0.31 (0.73) / **0.13 (0.51)**	−0.42 (0.69) /−**0.48 (0.53)**

^a^ Cells sum to 1. ^b^ Figures in bold pertain to girls; figures between parentheses are SD’s

In the bottom panel of the Table, the mean scores for resource control over the three assessment points for each of the joint trajectory groups are shown. An ANOVA was performed with mean resource control score serving as the dependent variable. Narcissism trajectory group, total bullying trajectory group, and gender were the between-subjects factors. Results revealed a main effect for bullying group: *F* (2, 365) = 26.40, *p* < 0.001. No other significant main or interaction effects emerged. Post-hoc multiple group comparisons using Tukey’s *d* showed that the scores of children in the highest bullying trajectory group were significantly higher than were scores for children in the medium bullying trajectory group (*p* < 0.001). In turn, these latter scores significantly exceeded those for children in the average bullying trajectory group (*p* < 0.001), and these children scored significantly higher than those in the low bullying trajectory group (*p* < 0.001). Taken together, across gender and level of narcissism, more intense bullying is associated with higher resource control.

## Discussion

The present multi-informant study examined longitudinal linkages between preadolescents’ narcissistic traits and two different forms of bullying. Moreover, we investigated to what extent children high in narcissism are successful in obtaining social dominance in the peer group, and how elevated bullying contributes to this outcome. An important conclusion of the present work, consistent with expectations, is that with respect to narcissism children do not constitute one homogenous group, neither in terms of mean scores, nor with respect to the linkages with bullying. Interestingly, contrary to previous work in which no gender differences emerged (Fanti and Kimonis 2012), marked differences between boys and girls were found with respect to the link between narcissism and bullying.

For girls, across both forms of bullying no systematic association with level of narcissism emerged, and girls were quite unlikely to be assigned to the highest bullying trajectory group. Relative to girls, boys were more likely to engage in high levels of bullying. More important for the present purposes, boys high in narcissism were substantially more likely to display high levels of both direct and indirect bullying than were their male peers lower in narcissism. This link with narcissism was stronger for indirect than for direct bullying. Although for boys being consistently high in narcissism is strongly associated with being consistently high in both types of bullying (as well as total bullying), in both instances the reverse pattern was not observed. Narcissistic boys were also quite successful in their pursuit of social dominance, but only when they engaged in high levels of bullying. Below we discuss our findings in more detail.

Using a person-centered approach, for all variables examined multiple-group trajectory models fitted the data best. Noteworthy, Fanti and Kimonis (2012) found a linkage between narcissism and more intense bullying, but they did not distinguish between potentially different trajectory groups of bullies or subgroups of narcissistic children. For most narcissism and bullying trajectory groups, mean scores were stable or changed only slightly over time. Only a relatively small number of children was assigned to the high narcissism trajectory group. This came as no surprise, given the almost normal distribution of narcissism scores (although somewhat positively skewed) and the mean score for this high narcissism trajectory group being more than 1 SD above the mean. Boys were moderately overrepresented in this group. In a similar vein, for both forms of bullying relatively few children followed the highest trajectory, with the large majority being male.

For boys, the joint analysis of the narcissism and bullying trajectories revealed a clear link between high narcissism and high direct bullying. In fact, boys following the high narcissism trajectory were more than twice as likely as their peers medium or low in narcissism to be assigned to the highest direct bullying group. Interestingly, relative to direct bullying, boys with high levels of narcissism were even substantially more likely to be assigned to the highest indirect bullying trajectory group. Importantly, variable-centered approaches such as regression analyses or SEM do not distinguish between different trajectory groups and can therefore not capture potential differences between these groups in terms of associations between variables.

One possible explanation for this difference as a function of bullying type is that narcissistic boys perceive indirect bullying as being more effective in obtaining their goals of power, dominance, and prestige. Alternatively, given the difficulties narcissists encounter when cooperating with others (e.g., Miller et al. 2007), it may also be that narcissistic boys preferentially engage in the kinds of (indirect) bullying they can most easily perform solitarily. In contrast, several forms of direct bullying require cooperating with peers to most effectively harass a victim. For instance, direct relational bullying often pertains to a victim being rejected or ostracized by a group, and direct possession-related bullying may be most effective when performed collectively (e.g., hiding or damaging a bicycle together, throwing around a schoolbag between multiple classmates).

While highly narcissistic boys were likely to be high on both direct and indirect bullying, these relationships were not symmetrical. In fact, the majority of the boys assigned to the trajectory with the highest bullying scores were not on the high narcissism trajectory. Hence, among boys high narcissism is one of more factors that predict intense bullying.

To the extent that narcissism involves the ongoing need to obtain admiration, and to feel powerful, it appears that highly narcissistic boys are quite successful in nourishing their grandiose self. Specifically, most – but not all - highly narcissistic boys scored above average in terms of resource control. However, our analyses strongly suggest that bullying, rather than narcissism as such, is the critical factor yielding high resource control. For instance, low narcissistic boys high on bullying were more successful in this regard than their high narcissistic peers low in bullying. Moreover, the few girls that showed high bullying also received high resource control scores, although they were not high in narcissism.

Noteworthy, high scores for resource control were also observed among youth medium or low in bullying, albeit less frequently. This finding is in line with the work of Vaillancourt and Hymel (2006), who noted “there are two different pathways to achieving status (visibility and influence) within the peer group, one through the explicit use of aggressive behavior, the other through the possession of peer-valued characteristics” (p. 398). Examples of these characteristics include being athletic or physically attractive.

Contrary to narcissistic boys, highly narcissistic girls were not more likely than their peers to engage in high levels of either direct or indirect bullying. In a similar vein, for girls no systematic overlap between high narcissism and high resource control was observed. These observations converge with findings reported by Salmivalli and colleagues (Salmivalli et al. 1999), who examined cross-sectional linkages between different dimensions of self-esteem and different participant roles in bullying situations (e.g., ringleader bully, defender) among adolescents aged 14–15. These authors found that “defensive egotism” (assessed with items such as “always wants to be the center of attention”; “can’t take criticism”) was positively associated with bullying for boys, whereas no such linkage was identified for girls.

What may account for this marked gender difference observed in two independent studies? Salmivalli et al. (1999) speculated that the bullying of boys is more strongly driven by dispositional traits (e.g., narcissism), whereas girls’ bullying is more contingent on situational and psychological factors such as being stimulated to bully by close friends or “clique” members. However, even if narcissistic girls are less likely than narcissistic boys to bully, how can they reconcile their grandiose sense of self and privileged status over others with their only average social dominance scores? One possibility is that narcissistic girls do long for admiration and prestige, but are somehow not successful. Alternatively, they may try to satisfy their self-motives of grandiosity and power in other, more communal domains. For instance, they may exaggerate their qualities in terms of being exceptionally (but instrumentally) kind and trustworthy (see Gebauer et al. 2012).

The present research has possible implications for intervention. To the extent that bullying provides narcissistic boys the position of dominance and power they aspire, they are not likely to refrain from this successful behavior. Currently, the treatment of narcissistic traits in youth is still in its infancy, and not much is known about the factors that cause and maintain these traits (Thomaes and Brummelman 2015). Hence, intervention is more likely to be effective when it seeks to reduce the rewards of bullying. The peer group is pivotal in this regard, given that bullies can only achieve dominance and prestige, when their actions are reinforced by peers. Interestingly, recent work has shown that the Finnish anti-bullying program “KiVa” effectively weakens the link between bullying and a dominant position in the peer group (Kärnä et al. 2011). The KiVa program aims to render bullying an unsuccessful strategy by focusing in particular on changing the behavior of uninvolved bystanders.

Useful extensions may be to teach narcissistic bullies that there are also other ways to achieve social dominance (e.g., by increasing athletic competence). Given that narcissistic individuals are typically low in empathy, and narcissism and psychopathy tend to co-occur (Thomaes et al. 2008; Van Baardewijk et al. 2008b), their bullying may be sustained by low sensitivity to signs of distress in others. Research has shown that making victims’ distress cues more salient, reduces aggression among children high in psychopathy (Van Baardewijk et al. 2008a). Hence, confronting narcissistic bullies more directly and explicitly with the misery they bring about might decrease their unwanted behavior.

## Limitations and Directions for Future Research

Our findings are based on primarily Caucasian pre-adolescents. To examine generalizability, future research should examine youth from a broader age range and other ethnic groups. Second, although a joint trajectory approach is well suited to prospectively examine the overlap of two constructs of interest, longitudinal designs do not permit causal inferences. Although it appears that among boys high narcissism leads to high bullying, it may also be that the rewards of bullying maintain or further elevate their narcissism. Third, our findings do not speak to the specificity of the results for narcissism, versus for instance the two other “dark triad traits” of psychopathy and Machiavellianism (Paulhus and Williams 2002), or general self-esteem (Fanti and Henrich 2015). Fourth, our person-centered approach also has disadvantages. For instance, individuals cannot be assigned to one of the distinct (latent) classes with perfect precision. Moreover, the presence of discrete groups is assumed, but the distribution of true scores may be continuous instead of discrete. Hence, latent classes do not necessarily correspond to truly existing different groups in the population. Finally, we did not examine children’s motivation to engage in different types of bullying, and it remains to be investigated why elevated narcissism among boys was stronger associated with indirect versus direct bullying. Future research is needed to investigate why children prefer certain types of bullying over others, and the role of narcissism in this regard.

Notwithstanding these limitations, the present study provides important new knowledge regarding the developmental linkages between narcissism, bullying and social dominance in youth. First, there are different trajectory groups of children who differ in level of narcissism. Second, whereas highly narcissistic boys show an elevated inclination to engage in high levels of bullying, boys low and medium on narcissism are (far) less likely to bully. Third, the link between high narcissism and high bullying is stronger for indirect versus direct bullying. Fourth, for girls higher narcissism is not associated with more frequent bullying. Finally, recent research among both adults and youth (e.g., Kuefner et al. 2013) has shown that many narcissists are at increased risk for peer rejection and isolation. Although the behavior of narcissistic youth is thus not necessarily interpersonally effective, it appears that the bullying of high narcissistic boys is instrumental in establishing social dominance in the peer group, which may serve to maintain or enhance their grandiose self.

## Electronic supplementary material

Below is the link to the electronic supplementary material.

ESM 1 (DOCX 66 kb)
