# Supplementary material for: Narcissism, Bullying, and Social Dominance in Youth: A Longitudinal Analysis
Source: J Abnorm Child Psychol. 2015 Feb 3;44(1):63–74. doi: 10.1007/s10802-015-9974-1 (PMC4715128; doi:10.1007/s10802-015-9974-1)
Supplement: Supplementary file 1 — (DOCX 66 kb) [file 10802_2015_9974_MOESM1_ESM.docx]

Table 1

*Correlations Among Study Measures at Time 1*

|  | Narcissism | Direct Bullying | Indirect Bullying | Total Bullying | Resource  Control |
| --- | --- | --- | --- | --- | --- |
| Narcissism |  | .17* | .12* | .17* | .12* |
| Direct Bullying |  |  | .71** | .96** | .41** |
| Indirect Bullying |  |  |  | .80** | .47** |
| Total Bullying |  |  |  |  | .46** |

*Note*. **p* < .05 ** *p* <.01

Table 2

*Correlations Among Study Measures at Time 2*

|  | Narcissism | Direct Bullying | Indirect Bullying | Total Bullying | Resource  Control |
| --- | --- | --- | --- | --- | --- |
| Narcissism |  | .05 | .04 | .09 | .04 |
| Direct Bullying |  |  | .71** | .96** | .47** |
| Indirect Bullying |  |  |  | .70** | .42** |
| Total Bullying |  |  |  |  | .49** |

*Note*. **p* < .05 ** *p* <.01

Table 3

*Correlations Among Study Measures at Time 3*

|  | Narcissism | Direct Bullying | Indirect Bullying | Total Bullying | Resource  Control |
| --- | --- | --- | --- | --- | --- |
| Narcissism |  | .12* | .07 | .11 | .12* |
| Direct Bullying |  |  | .67** | .95** | .46** |
| Indirect Bullying |  |  |  | .79** | .39** |
| Total Bullying |  |  |  |  | .46** |

*Note*. **p* < .05 ** *p* <.01

|  |  |
| --- | --- |
|  |  |

*Figure 1*. Mean narcissism and bullying scores at three time points, for each trajectory group.
